# Development and Application of Tetra/Penta-Nucleotide SSR Markers for Paternal Identification in the Tea Plant (*Camellia sinensis)*

**DOI:** 10.3390/plants14223500

**Published:** 2025-11-17

**Authors:** Yingqi Liu, Kailing Chang, Yaning Zhu, Dandan Tang, Wei Chen, Qian Tang, Liqiang Tan

**Affiliations:** 1 College of Horticulture, Sichuan Agricultural University, Wenjiang District, Huimin Road, No. 211, Chengdu 611130, China; lyq0072025@163.com (Y.L.);; 2 Tea Resources Utilization and Quality Testing Key Laboratory of Sichuan Province, Chengdu 611130, China

**Keywords:** tea plants, parentage analysis, SSR markers, breeding

## Abstract

In this study, twenty-seven novel SSR markers derived from tetranucleotide or pentanucleotide repeat sequences were developed for tea plants (*Camellia sinensis*). These markers, along with three previously reported pentanucleotide SSR markers, were assessed for their polymorphisms and capabilities for parental analysis. Analysis of 48 tea cultivars revealed 142 alleles with an average polymorphic information content (PIC) of 0.44, confirming the high polymorphism of these markers. Meanwhile, the capability of these markers for paternal identification in tea plants was also validated. Theoretical calculations yielded a cumulative exclusion probability (CPE) over 99.9999%. In the analyses of real samples using the exclusion method, among eight samples with confirmed parent–offspring relationships, both pair- and trio-mismatch counts were ≤1, whereas non-paternal samples exhibited average pair- and trio-mismatches of 5.13 and 7.96, respectively. When assessed by the combined parentage index (CPI), all trio-CPI values for confirmed parents–offspring exceeded 10^4^ (average: 1.18 × 10^7^), while the average trio-CPI for combinations with correct maternal but incorrect paternal parents remained below 10^−2^. Finally, using this set of markers, we successfully identified 41 offspring derived from reciprocal crosses with open pollination between ‘Fuding Dabaicha’ and ‘Chuancha Erhao’. Their trio-mismatches with the parent pair were ≤1, while trio-CPI exceeded 10^4^, meeting the established criteria for parentage relationships.

## 1. Introduction

As one of the most popular beverage plants, the tea plant (*Camellia sinensis* (L.) O.Kuntze) is widely cultivated with about five million hectares around the world [1]. It is an evergreen, perennial, outcrossing woody plant. Traditionally, the breeding of tea plants primarily relies on selecting superior individuals from existing sexual population resources (also called “systematic selection”), or selecting from materials created through natural or artificial hybridization. The pedigree relationships between tea cultivars were rarely known, unless they resulted from artificial hybridization. However, as clonal tea cultivars (propagated through cuttings) become more numerous and increasingly replace sexual populations, more and more new tea cultivars are being developed through crossbreeding between clonal tea cultivars [2]. This creates conditions for constructing the pedigree relationships among these new tea cultivars.

For breeding, a clear pedigree relationship is very useful, including the following: gaining a detailed understanding of the genetic background of each cultivar, which helps to understand the inheritance patterns of specific traits; optimizing hybridization strategies and creating more effective crossbreeding plans, avoiding the adverse consequences of inbreeding; and aiding in protecting germplasm resources and in gene-mapping research [3,4]. In addition, clear pedigree records also provide evidence for intellectual property protection of parental lines and new cultivars [5].

Leveraging the self-incompatibility characteristic of tea plants, a large amount of hybrid breeding materials can be created through natural crosses between elite clonal cultivars [6]. Natural crossing is also a necessary choice because artificial hybridization of tea plants consumes a lot of time and labor, and the fruit-setting rate is very low [7]. Many registered tea cultivars are derived from natural crossbreeding of clonal cultivars, such as ‘Zhenong 117’ and ‘Fuyun 10 hao’ [8].

Although natural crosses save labor, they introduce a new problem: only the maternal parent of the new hybrids is known, while the paternal relationship remains unclear. To address this problem, breeders have attempted to use various types of DNA markers for parentage identification in tea plants [9]. As early as 2001, Li et al. used the random amplified polymorphic DNA (RAPD) markers to conduct paternity testing on the F_1_ generation plants obtained from the hybridization of *C. sinensis* and *C. ptilophylla* [10]. Tan et al. [11] employed simple sequence repeat (SSR) markers to analyze the pedigree relationships among 128 tea plant cultivars. Single-nucleotide polymorphism (SNP) markers based on high-throughput sequencing have also been used for parental identification in open-pollinated tea plant populations [12]. Nevertheless, among different types of DNA markers, SSRs (also called short tandem repeats (STRs)) are the first choice for parental identification due to their high polymorphism, reliability, and cost-effectiveness. In humans and some animals (like horses and cattle), Chinese national standards for paternity testing based on SSR markers have been established and are widely used in forensic and breeding practices.

SSRs consist of tandem repeat sequences made up of one to six nucleotide units and are prevalent in eukaryotic genomes, often evenly distributed. They are relatively easy to mutate with unique mutation characteristics, such as the addition or reduction in repeat units, making them ideal molecular markers [13]. Particularly, tetranucleotide and pentanucleotide SSRs (tetra/penta-SSRs), which have good stability and ease of genotyping, are better genetic markers for parentage analysis [14]. Although many SSR markers have been developed for tea plants, those based on tetra/penta-SSRs are still relatively scarce. In addition, in previous studies on parentage analysis of tea plants, the main basis for judgment relied on the clustering analysis or the number of mismatched loci that did not conform to Mendelian inheritance patterns [15]. However, the rigor of this basis for judgment is still far from sufficient.

Therefore, the purpose of this study is to develop a set of tetra/penta-SSR markers for parentage testing in tea plants, with the aim of providing pedigree support for the open-pollination hybrid breeding of tea plants. To achieve this goal, we first analyzed the distribution of tetra/penta-SSRs in the tea genomes, then experimentally screened hundreds of primer pairs to obtain high-quality markers and conducted genotypic analysis on common tea cultivars to acquire markers’ polymorphism and allele frequencies. Then, we performed analyses among samples with known pedigree information to test whether these markers met the requirements for determining or excluding parentage relationships. Finally, an empirical application was conducted to identify the paternal parents of tea individuals derived from open pollination between ‘Fuding Dabaicha’ and ‘Chuancha Erhao.’

## 2. Results and Analysis

### 2.1. Tetra/Penta-SSR Loci Distribution in the Plant Genomes

**Figure 1:**
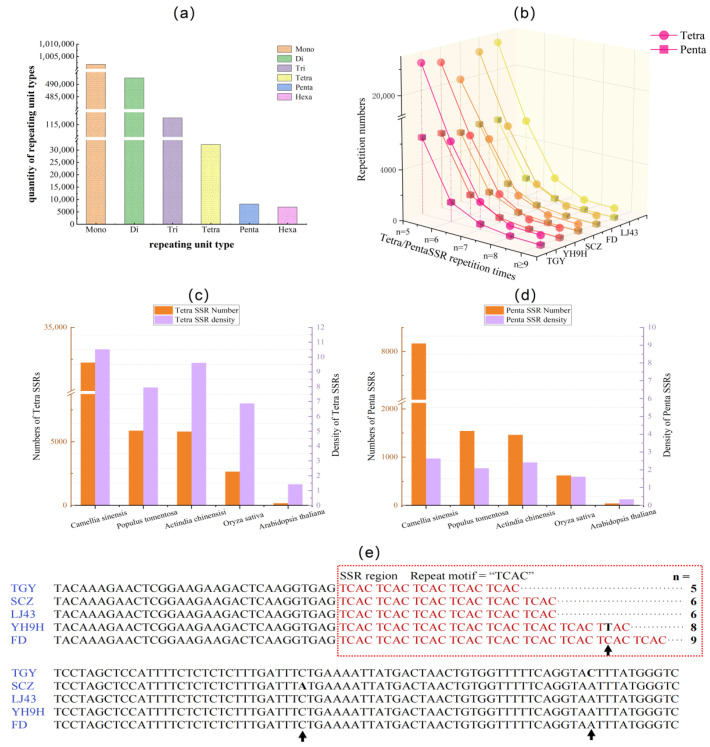
The distribution and variation in SSRs in the tea and other plant genomes. (**a**) The distribution of different types of SSRs in the genome of ‘Tieguanyin’. (**b**) The number of tetra- and penta-SSRs in the genomes of different tea cultivars (TGY ‘Tieguanyin’, YH9H ‘Yinghong 9 hao’, SCZ ‘Shuchazao’, FD ‘Fudingdabaicha’, LJ43 ‘Longjing43’). (**c**,**d**) Total number and density of identified tetra- and penta-SSRs in *Camellia sinensis*, *Oryza sativa*, *Arabidopsis thaliana*, and their flanking sequences in different tea cultivar genomes. The repeat motif and repeat number (n) were shown, and the arrows indicated the presence of an SNP mutation. (**e**) The sequence alignment of TGY, SCZ, LJ43, YH9H, and FD, marked with *CsTetra*11. The repeated units are highlighted in red boxes, where “n” represents the number of repeats and arrows indicate mutation sites within the sequence alignment.

In the ‘Tieguanyin’ genome, there are 32,227 tetra-SSR loci and 8165 penta-SSR loci with a repeat number of five or more. This number was significantly less than trinucleotide SSRs (117,679, Figure 1a). Across five different tea plant cultivar genomes (TGY, SCZ, FD, LJ43, and YH9H), the number of tetra-SSRs varied from 28,450 to 32,227, while penta-SSRs remained varied from 7287 to 8165. The SSR number decreased rapidly as the repeat number increased in all analyzed tea plant genomes (Figure 1b). Among the five studied plant species (Figure 1c,d), *Arabidopsis* showed the lowest densities of tetra- and penta-SSRs (1.42/Mb and 0.34/Mb, respectively), followed by rice (6.87/Mb and 1.61/Mb), poplar (7.90/Mb and 2.08/Mb), and kiwi (9.60/Mb and 2.41/Mb). All were significantly lower than in tea plants (10.52/Mb and 2.63/Mb). These results suggest that tetra- and penta-SSRs are rich and relatively stable across different tea plant cultivars.

To investigate the mutational characteristics of SSRs, we analyzed the polymorphism of a randomly selected tetra-SSR locus across five tea cultivars (Figure 1e). This sequence exhibited significant length variations due to changes in the copy number of its repeat unit: five in TGY, six in SCZ/LJ43, and eight/nine in YH9H/FD. Additionally, although SNPs were identified both within the SSR motif and in the flanking regions (Figure 1e), they did not affect the length of the PCR amplicons. These features make it an ideal candidate for developing parentage testing markers.

### 2.2. Tetra/Penta-SSR Markers Development for Tea Plants

**Figure 2:**
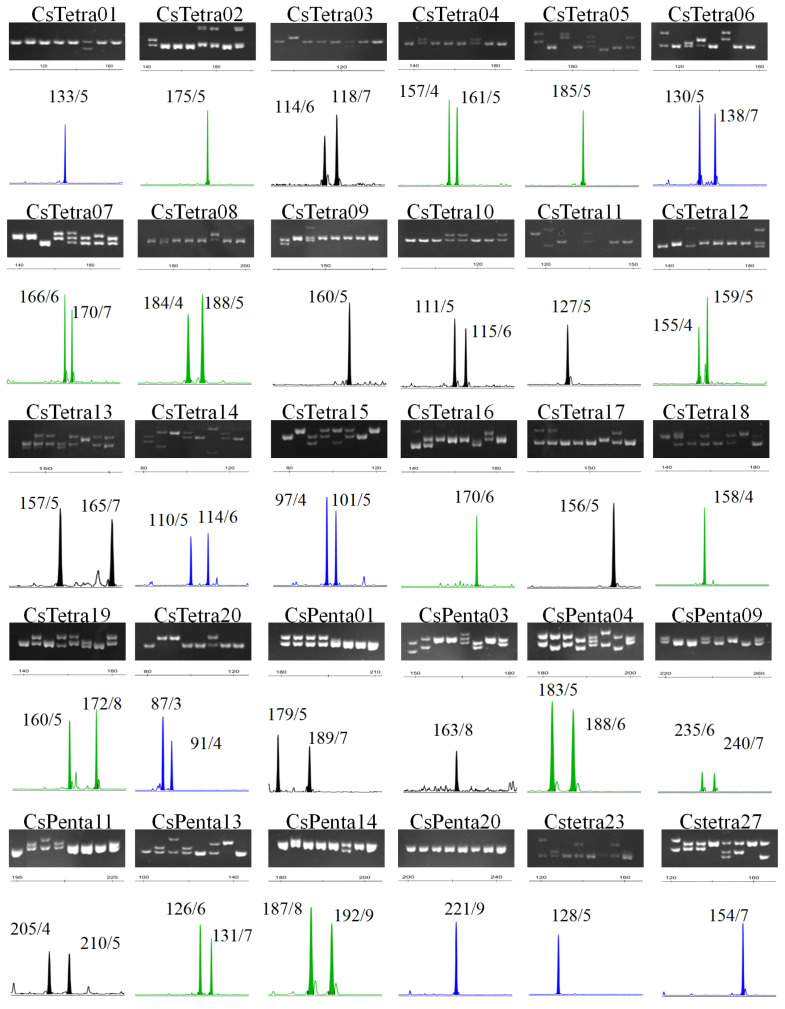
Electropherograms from polyacrylamide gel and capillary electrophoresis showing the 30 screened tetra/penta-SSR markers. Beside each peak in the electropherogram lies the PCR product size and the inferred repeat number of the SSR unit.

More than 200 pairs of primers were designed based on tetra/penta-SSR loci in the tea plant transcriptome sequence. These primers underwent initial screening via PCR amplification and polyacrylamide gel electrophoresis, followed by further selection using capillary electrophoresis. Among them, 27 pairs demonstrated outstanding performance: they exhibited high amplification success rates, produced clear PCR products of the expected size, and contained at least two easily distinguishable alleles per locus (Figure 2). These 27 primer pairs were selected as high-quality molecular markers. Additionally, three previously published penta-SSR markers, which have been extensively used by our research group, were incorporated. The final set of markers used for subsequent analyses comprised 20 tetra-SSR and 10 penta-SSR markers (Figure 2 and Table 1). These markers were distributed across the 15 chromosomes of tea plants, with the exception of chr06.

### 2.3. Marker Polymorphism

Forty-eight tea plant accessions were analyzed using the 30 SSR markers to assess genetic diversity; results are presented in Table 2 (for genotype see Supplementary Materials, Table S4). These markers resolved a total of 142 alleles, averaging 4.7 per locus (range = 2–10). The mean polymorphic information content (PIC) score across all markers was 0.442. Among individual loci, CsTetra05 was the most informative, revealing 10 alleles and achieving a PIC of 0.814. Thirteen markers displayed PIC values > 0.5, signifying moderate-to-high polymorphism. The size of the fragment ranges from 87 to 240, the range of repeated units is from 1 to 11, and there are imperfect repeated units. These 142 allele frequencies ranged from 0.01 to 0.98, averaging at 0.211.

### 2.4. Parental Relationship Analysis Using Samples with Known Parents

With a cumulative exclusion probability (CPE) of (1 − 4.16 × 10^−17^)—well above the required benchmark of 99.9999%—this set of 30 SSR markers demonstrated high potential for parentage assignment. We validated their performance using eight hybrid offspring from the cross EW × CM217 alongside 48 candidate parental plants (Table 3 and Supplementary Materials, Table S5). All offspring perfectly matched their documented parents (EW: mother; CM217: father), showing zero pair- or trio-mismatches. Their trio-based CPI scores surpassed the critical threshold of 10^4^, ranging between 1.12 × 10^5^ and 1.18 × 10^6^. In negative controls where unrelated individuals served as putative fathers (n = 46), offspring displayed significant genetic discrepancies: mean pair mismatches = 5.13 (range: 0–11); mean trio-mismatches = 7.96 (range: 1–14; Table 3). Notably, even the highest CPI value involving a false paternal candidate did not exceed 0.007, which is dramatically lower than the forensic exclusion cutoff of 0.02.

Taken together, this penta/tetra-SSRs panel can reliably confirm biological fathers in tea-breeding programs and effectively excludes unrelated males when maternal information is known, achieving diagnostic accuracy through pair- and trio-mismatch and CPI scoring.

### 2.5. Analysis of Results for Unknown Parental Materials

We assessed the utility of these markers for paternity verification in open-pollinated tea breeding using 80 seedlings originating from seeds produced by neighboring clonal plants of FD and CC; see Supplementary Materials Table S5 for genotypes. As summarized in Table 4, reciprocal cross analyses confirmed no mismatches between offspring and their known mothers (FD/CC). Specifically, 13 out of 30 FD-seedlings traced back to CC pollen, while 28 of 30 CC-seedlings were sired by FD. True parent–offspring trios displayed robust genetic consistency (≤1 trio-mismatch; CPI > 10^4^, range: 1.10 × 10^4^–2.12 × 10^8^). Testing against 46 unrelated controls yielded trio-mismatch counts of 2–15 (means: 8.31 and 7.41), effectively ruling out false paternal claims. The method also resolved alternative paternity cases for 15 seedlings (e.g., sired by EW), though details are omitted here.

## 3. Discussion

### 3.1. High Abundance of Tetra/Penta-SSRs in the Tea Plant Genome

Tetra/penta-SSRs represent less common classes of SSRs in plant genomes. For example, among 22 species of Lythraceae, the number of tetra-SSRs ranges from 6 to 12, and penta-SSRs range only from 0 to 2 [16]. In this study, we compared the distribution of tetra- and penta-SSRs across various plant genomes and within five tea cultivar genomes using a unified criterion (repeat unit length n ≥ 5). The tea plant genome contains significantly more tetra- and penta-SSRs than other species—both in total number and density—with densities reaching 9.11 per Mb for tetra-SSRs and 1.95 per Mb for penta-SSRs, respectively. This abundance likely correlates with its large genome size (>3 Gb) and exceptionally high proportion of repetitive sequences (>80%) [17]. Repetitive and non-coding regions tend to accumulate SSR motifs. Furthermore, tetra/penta-SSR loci numbers are conservative among different tea cultivars, providing a robust basis for developing molecular markers. Additionally, increasingly available genomic data from diverse tea plants enhance our capacity to design SSR markers, predict polymorphism levels, analyze variation patterns, and conduct related studies [18].

### 3.2. Criteria for Molecular Markers in Parentage Analyses

Compared to other genetic studies, parentage analysis imposes stricter requirements on molecular markers. These requirements include the following: a balance between genetic stability and variability, codominant expression, ease of detection, and wide genomic distribution and uniformity to minimize linkage disequilibrium effects [19]. Significantly, the tetra- and penta-SSR markers developed in this study meet all these criteria and are, therefore, suitable for tea plant parentage verification. Those tetra/penta-SSR loci that exhibit moderate polymorphism are codominantly inherited, ensure accurate Mendelian transmission to offspring, and maintain sufficient mutation rates for adequate CPE and CPI values [20,21]. Moreover, empirical evidence confirms the feasibility of acquiring sample genotypes via both polyacrylamide gel and capillary electrophoresis systems. Therefore, we conclude that these genetic markers constitute a viable tool for parentage assignment in tea plant breeding.

### 3.3. Parentage Determination Criteria in Tea Plants

In human paternity testing, parentage determination typically depends on two criteria: the CPI value and the count of mismatched loci. Generally, a CPI exceeding 10,000 is required, though permissible mismatch thresholds vary with the number of tested loci. For example, no mismatches are allowed for 15 markers, ≤1 trio-mismatch is permitted with 19 loci, and ≤2 trio-mismatches are accepted for 28 loci. Typically, ≥3 pairwise mismatches or ≥4 trio-mismatches lead to exclusion of parentage [22]. In this study, we evaluated eight tea plant samples with known parent–offspring relationships using 30 tetra- and penta-SSR markers. Results showed actual CPI values reached 10,000 with zero pair/trio-mismatches in true pairs (Table 3). Non-parental trios exhibited significantly increased average trio-mismatches (7.96) and markedly lower CPI values (<0.02). These findings demonstrate our marker set effectively discriminates between true and false parent–offspring relationships in tea plants. The human paternity testing standards can serve as reference benchmarks for parentage analysis in tea plants. Indeed, the tea plant genome shares notable similarities with the human genome—including comparable size (~3 Gb), predominant diploidy, and high heterozygosity—resulting in analogous genetic transmission patterns between parents and progeny. Accordingly, our proposed standards for tea plant parentage testing include the following: using 15~30 SSR markers, trio-mismatches ≤ 1, and CPI ≥ 10,000.

### 3.4. The Application Scope and Limitations

The above results validate that the tetra/penta-SSR markers can effectively identify paternal contributors from naturally pollinated tea plants, particularly when pollen originates from a limited pool of clonal cultivars. Accordingly, we propose the following breeding protocol for tea plants: (i) Create dedicated hybridization gardens with genetically diverse elite clones or repurpose existing comparative trial plantations of clonal cultivars; (ii) conduct annual seed harvesting accompanied by recording of maternal sources and candidate pollen donors; (iii) cultivate the seedlings and evaluate their phenotypic performance; and (iv) the identified elite seedlings will undergo SSR-based parentage assignment using the markers described herein, enabling precise lineage tracing.

Acknowledging certain constraints is essential. Firstly, the 48 tea plants utilized for marker polymorphism analysis and allele frequency estimation may fail to fully represent the genetic diversity of the tea germplasm in China, potentially compromising the accuracy of PI/CPI metrics. This limitation can be addressed in subsequent studies by progressively incorporating additional genotype data and refining allele frequency estimates. Secondly, accurate paternity determination becomes challenging when seed-collected tea plants are surrounded by numerous diverse tea cultivars or sexually reproductive populations [23]. Thirdly, complex mating dynamics in tea plants—such as consanguineous breeding, somatic mutations induced by prolonged vegetative propagation within clonal cultivars, and overlapping generations—may introduce uncontrollable confounding factors into parentage analyses [24]. Consequently, robust paternity assignment for novel tea plants requires synergistic integration of molecular marker data and comprehensive analyses of potential pollen sources within the maternal environment.

## 4. Materials and Methods

### 4.1. Plant Materials and DNA Extraction

This study utilized three different types of plant materials for various purposes, categorized as follows. For marker development, eight tea cultivars were employed: ‘Ziyan’ (ZY), ‘Emei Wenchun’ (EW), ‘Chuancha Erhao’ (CC), ‘Chuanmu 217’ (CM217), ‘Chuancha 28’ (CM28), ‘Zijuan’ (ZJ), ‘Huangjinya’ (HJY), and ‘Fuding Dabai cha’ (FD). For the analysis of marker polymorphism and allele frequencies, 48 tea plant cultivars were used, and the details are shown in Supplementary Materials Table S1. To test the capability of the developed markers for paternal identification, eight offspring of EW × CM217 were used. Finally, as a practical application of parentage testing, 80 seedlings originating from open-pollinated seeds of adjacently cultivated FD (30) and CC (50) plants were sampled for analysis.

These plants were grown at either the tea plant experimental field of Sichuan Agricultural University (Wenjiang, Chengdu, China) or the tea plant breeding center of Sichuan Agricultural University (Yaan, Sichuan, China). Young leaves were collected from previously mentioned tea plants and then rapidly frozen with liquid nitrogen and storage at −80 °C before DNA extraction. Genomic DNA was extracted using a modified CTAB reagent kit method (Tiangen). The DNA concentration was then diluted to approximately 50 ng/μL for use.

### 4.2. SSR Loci and Marker Primer Design

The chromosome-level genome sequence of tea plants was downloaded from TPIA (http://tpia.teaplants.cn) and the Tea Pangenome Data website (https://www.tea-pangenome.cn/), while the whole genome sequences of rice (*Oryza sativa*), Arabidopsis (*Arabidopsis thaliana*), poplar (*Populus* L), and kiwi fruit (*Actinidia chinensis*) were downloaded from NCBI. We used MISA-Perl(http://pgrc.ipk-gatersleben.de/misa/) to search the tetra/penta-SSR loci with the repeat number ≥5 in the previously mentioned genomes.

For marker development, the tetra/penta-SSR loci from the tea plant transcriptome sequence [25] were used, and PCR primers were designed by Primer 3.0. The primer length was set to 18–20 bp, and the PCR product size was aimed to be between 100 and 250 bp. The designed primers were synthesized by Shenggong Biological (Chengdu, China) Company.

### 4.3. PCR Amplification and Allele Detection

PCR amplification [26] was performed using a 10 µL reaction system: 2.7 µL of double-distilled water; 5 µL of 2× Accuproof HiFi HotStart SuperMix; 0.4 µL of Primer-F (concentration 10 µmol); 0.4 µL of Primer-R (concentration 10 µmol); and 1.5 µL of DNA template (concentration 30 ng/µL). The PCR amplification reaction program was as follows: Step 1: Pre-denaturation at 94 °C for 4 min; Step 2: Denaturation at 94 °C for 30 s; Step 3: Annealing at 56 °C for 30 s; Step 4: Extension at 72 °C for 40 s; Step 5: Repeat Steps 2–4 for a total of 35 cycles; and Step 6: Final extension at 72 °C for 10 min; hold the products at 4 °C. The amplified products were analyzed using 7% polyacrylamide gel electrophoresis (PAGE) at 120 V for 90 min. After electrophoresis, the gel was stained in 0.01% nucleic acid dye for 15 min, followed by visualization and image capture for documentation. Primer pairs that yield clear target amplification products and exhibit high polymorphism are selected for further analysis. The selected markers are named in the following manner: starts with “*Cs*” (*Camellia sinensis*), followed by “Tetra” or “Penta”, and a two-digit number; for example, *CsTetra01*. Among them, *CsPenta01*, *CsPenta03*, and *CsPenta04* are from Tan et al. [27].

To achieve higher resolution genotyping results, we use fluorescent-labeled primers combined with capillary electrophoresis for subsequent analyses. The selected primers (upstream) are labeled with one of the three types of fluorescent dyes (FAM, TAMRA, or HEX). These labeled primer pairs are used to amplify DNA from the previously mentioned plant materials. The PCR products are then subjected to capillary electrophoresis using the ABI 3730XL DNA sequencer (Applied Biosystems, USA, California).

### 4.4. Data Collection and Analysis

The alleles were represented by the repeat number of the target tetra/penta-SSRs, which was calculated according to the reference sequence and the detected sizes. For irregular variations, if the size difference from the standard allele was within 1.5 bp (typically attributed to detecting errors or ±1 bp indels in the flanking sequences), they were considered the same allele. If the size difference exceeded 1.5 bp compared to adjacent standard alleles, they were classified as sub-alleles and labeled by appending a letter “a” after the repeat number. As tea plants are diploid, if only one allele is detected at a locus for a sample, it is considered to be in a homozygous genotype. If three alleles are detected, it is common to select the two alleles with the highest peaks to form a heterozygous genotype. In subsequent validations, experiments can be repeated to confirm the results, or other molecular markers (such as SNPs) can be used for further confirmation.

The results of the genotype analysis are imported into GeneAlex 6.5 software [28] to calculate the number of alleles (*N_A_*), observed heterozygosity (*H_O_*), expected heterozygosity (*H_E_*), and polymorphism information content (*PIC*). Parentage analysis for known and unknown offspring is performed using CERVUS 3.0.7 [29]. The pair mismatch (MM2) and triplet mismatch (MM3) were calculated by Cervus 3.0.

The probability of exclusion (PE) and cumulative probability of exclusion (CPE) are calculated with the following formulas:

$$PE=\sum_{i=1}^{n}p_{i}^{2}\times {\left(1-P_{i}\right)}^{2}+\sum_{i=1}^{n-1}\sum_{j=i+1}^{n}2\times p_{i}\times p_{j}\times (1-p_{i}-p_{j}{)}^{2}$$

where n is the number of alleles, *p_i_* is the frequency of allele *i*, and *p_j_* is the frequency of allele j:

$$CPE=1-\Pi_{i=1}^{k}\left(1-PE_{k}\right)$$

where *k* is the number of genetic markers in the detection system.

The parentage index (PI) and combined parentage index (CPI) are calculated using Excel software with formulas [30] shown in Supplementary Materials Table S2.

## 5. Conclusions

In this study, we developed a novel SSR (simple sequence repeat) marker system based on tetranucleotide and pentanucleotide loci, effectively validating its feasibility for parental identification in tea trees. This advancement not only offers significant molecular tools for future genetic improvement and conservation of tea resources but also enhances the precision of breeding practices. With the ongoing evolution of breeding technologies, this marker system is poised to deliver substantial value in enhancing the genetic quality and overall breeding outcomes of tea trees.

## Figures and Tables

**Table 1:** The primer sequences, repeat motifs, and chromosomal locations of the 30 screened tetra/penta-SSR markers.

Marker ID	Forward Primer Sequence	Reverse Primer Sequence	Motif	Chromosome
*CsTetra01*	AAGTGCTAATTTGCTCGG	AAACCCTAGTCCCAGTAA	(TTAA)5	1
*CsTetra02*	GGCAGATCCAAGAAGGTG	AACAGAGTTGCTCGGAAG	(ATCA)6	2
*CsTetra03*	ACACGAGTTCTTTGGATT	CGTTACAACACCGATAGT	(TTAT)6	11
*CsTetra04*	TCAGGTGATGTCAAGCAT	TCCATCCTAATCAAACTAAT	(AAAT)5	2
*CsTetra05*	TTTCCCTACAAACTAAGAC	TGAGCACCCACTACATTC	(TTTA)5	4
*CsTetra06*	GTGTTCTGGGTTATCTTT	AATCCATTGCTAGTAGTGT	(GTTT)5	5
*CsTetra07*	GCTATAAACCCCAACCAA	GTCTGCATCATCAACCATAT	(ATAG)7	12
*CsTetra08*	ACAACACTCTTGGGTTCC	TGTGGGGTAATTCAAATC	(TTTA)5	15
*CsTetra09*	AATAGGTGGACAGGTGAG	TGATCCCAATATGATGTG	(ATAA)5	11
*CsTetra10*	TTCACTGCTTATGCTTGT	CAGGGTTATCCCTCACTA	(TATT)5	4
*CsTetra11*	TACAAAGAACTCGGAAGA	GACCCATAAATTACCTGA	(TCAC)5	10
*CsTetra12*	TTTCTGTTTTAATTCCCTTTGC	GGGTTGGTTTTGCCCTCT	(TGTT)5	8
*CsTetra13*	AAACCAAATCCACCATCT	AAGGGAATCTACTTGAATGA	(TTTG)5	9
*CsTetra14*	AGGTGGTAACAAGTCTCA	AAGTCACAAGCAGCAAAG	(TATC)7	2
*CsTetra15*	TGGGATGAGACAAAGGAG	GGGGAGGAGTTGAAATATGAAA	(GATG)6	13
*CsTetra16*	TATTTTCCGTTTGTTCTAAG	ACATCCAAACCAACCTCT	(TTTC)6	12
*CsTetra17*	TCTTGTCTCGTCGCTTCT	CAATTCATTACATCAAACCC	(ATCT)5	9
*CsTetra18*	TGTGCTCTGTTCTTGGTA	ATGAAGTAAAATAGGTCC	(ATTT)5	12
*CsTetra19*	ATTTCCAGCAATCCGTCG	CAATGCCAATAAAACAACAA	(ATGT)8	5
*CsTetra20*	TTGCTTCCACCGACAGAT	AGGGCAGGGCATAGATAA	(ATTT)5	13
*CsPenta01*	GAAGCGCATCTTTAACAGCC	ACAAAATGGAGACCGACCAC	(TGATG)5	14
*CsPenta03*	GAAGAACAAGTGTCGGGGAA	TCGGAGGTACAAAACTCATCTC	(GAGTT)5	4
*CsPenta04*	ACTCACACAAACCCGAAAGG	GGTTCAAATCAAGCTCGCTC	(GAAAA)6	5
*CsPenta09*	GCTGTGGACTTCGTAATCTGTT	GCAGCACTGTCCTTGATGG	(CCATA)5	4
*CsPenta11*	GTGCCACTGCTCTCCAACT	AACTGAATCGAGTCCGACCTTA	(CAACC)5	7
*CsPenta13*	GCTTCTCCTAGAGCAACATCTT	TTGGCTGGGTCATCTGGTTT	(CAAAC)6	9
*CsPenta14*	AGGAGGAGGAGGATGGAAGG	GCTTGTCAGGCATCACTCTAAG	(GAGGA)5	10
*CsPenta20*	GTTCCACTTAGCCCATCA	ATCCCAATCCAAACCAAA	(TGAGT)5	1
*CsPenta23*	CTAAATCCTTAGGTATCC	ATCTCAACAATTATCTGC	(TTTTG)5	5
*CsPenta27*	TCAGAACTTGGTGAACTCA	GGATTGGAGATGCTCTTAG	(TTTTG)5	3

**Table 2:** Polymorphism analysis results of the 30 SSR markers.

Marker	*N_A_*	*Repeat Number (n)*	*N_E_*	*I*	*H_O_*	*H_E_*	*PIC*
*CsTetra01*	2	3,5	1.066	0.141	0.064	0.062	0.06
*CsTetra02*	4	3,5,6,8	1.776	0.799	0.521	0.437	0.388
*CsTetra03*	2	6,7	1.045	0.107	0.044	0.043	0.043
*CsTetra04*	2	4,5	1.112	0.208	0.106	0.101	0.096
*CsTetra05*	10	1a,2,3,4,5,5a,6,7,9,11	6.019	1.967	0.851	0.834	0.814
*CsTetra06*	6	4,5,6,7,9,9a	2.147	1.091	0.354	0.534	0.503
*CsTetra07*	6	3,4,4a,6,7,8	3.125	1.297	0.667	0.68	0.627
*CsTetra08*	4	4,5,6,7	1.295	0.497	0.25	0.228	0.218
*CsTetra09*	3	3,4,5	1.411	0.565	0.083	0.291	0.272
*CsTetra10*	3	4,5,6	1.922	0.789	0.383	0.48	0.405
*CsTetra11*	5	4,5,6,8,9	3.238	1.316	0.25	0.691	0.636
*CsTetra12*	2	4,5	1.958	0.682	0.313	0.489	0.37
*CsTetra13*	5	4,5,6,6a,7	2.079	0.982	0.479	0.519	0.475
*CsTetra14*	7	2,3,4,5,6,7,9	3.856	1.522	0.771	0.741	0.702
*CsTetra15*	6	2,3,4,5,6,7	2.69	1.188	0.708	0.628	0.56
*CsTetra16*	6	2,4,5,6,7,8	3.103	1.334	0.583	0.678	0.628
*CsTetra17*	5	5,6,7,8,9	1.384	0.617	0.238	0.277	0.266
*CsTetra18*	6	1,2a,4,5,6,7	2.225	0.934	0.638	0.55	0.45
*CsTetra19*	8	2,3,4,5,6,7,8,9	3.093	1.417	0.688	0.677	0.637
*CsTetra20*	2	3,4	1.311	0.4	0.225	0.237	0.209
*CsPenta01*	3	5,6,7	2.542	1.013	0.708	0.607	0.538
*CsPenta03*	4	5,6,7,8	2.621	1.068	0.689	0.619	0.54
*CsPenta04*	5	3,4,5,6,7	2.848	1.306	0.787	0.649	0.613
*CsPenta09*	4	4,5,6,7	1.905	0.816	0.563	0.475	0.403
*CsPenta11*	4	4,5,6,6a	1.357	0.534	0.298	0.263	0.245
*CsPenta13*	5	6,7,8,9,10	2.586	1.205	0.463	0.613	0.573
*CsPenta14*	5	5,6,7,8,9	1.398	0.626	0.298	0.285	0.272
*CsPenta20*	4	5,6,7,9	2.064	0.942	0.542	0.515	0.468
*CsPenta23*	6	4,5,6,6a,7,8a	2.07	1.046	0.354	0.517	0.482
*CsPenta27*	8	1,2,3,4,5,6,7,8	5.085	1.926	0.684	0.803	0.778

Note: *N_A_* is the number of detected alleles, *N_E_* is the number of effective alleles, *I* is Shannon’s information index, *H_O_* is the observed heterozygosity, *H_E_* is the expected heterozygosity, and *PIC* is the polymorphic information content.

**Table 3:** Pair/trio-mismatching number and CPI values between real or unrelated parents and the eight offspring from EW × CM217.

Indicators for Parent–Child Relationships	Minimum	Maximum	Mean
Pair mismatches with female parent (EW)	0	0	0
Pair CPI with female parent (EW)	7.31 × 10^4^	3.06 × 10^7^	1.49 × 10^7^
Pair mismatches with male parent	0	0	0
Pair CPI with male parent (CM217)	1.16 × 10^3^	2.72 × 10^5^	5.45 × 10^4^
Trio-mismatches with the two real parents	0	0	0
Trio-CPI with the two real parents	1.12 × 10^5^	5.5 × 10^6^	1.18 × 10^6^
Pair mismatches with 46 accessions except the real parents	0	11	5.13
Trio-mismatches with EW and 46 unrelated male parents	1	14	7.96

Note: EW: ‘Emeiwenchun’, CM217: ‘Chuanmu217’, and CPI: combined parentage index.

**Table 4:** Paternal identification results for open-pollinated offspring tea plants using 30 SSR markers.

Indicators for Parent–Child Relationships	FD × CC (n = 13)			CC × FD (n = 28)		
	Minimum	Maximum	Mean	Minimum	Maximum	Mean
Pair mismatches with female parent	0	0	0	0	0	0
Pair CPI with female parent	1.11 × 10^3^	1.27 × 10^5^	2.51 × 10^4^	9.24 × 10^1^	6.45 × 10^6^	8.34 × 10^5^
Pair mismatches with male parent	0	0	0	0	0	0
Pair CPI with male parent	5.6 × 10^3^	1.67 × 10^7^	2.17 × 10^6^	1.14 × 10^2^	2.79 × 10^5^	2.02 × 10^4^
Trio-mismatches with the two real parents	0	1	0.23	0	1	0.18
Trio-CPI with the two real parents	1.10 × 10^4^	2.12 × 10^8^	2.64 × 10^7^	1.27 × 10^4^	3.68 × 10^7^	2.17 × 10^6^
Pair mismatches with 46 accessions except the real parents	0	12	4.54	0	11	4.42
Trio-mismatches with real female parent and 46 unrelated male parents	2	15	8.31	2	14	7.41

Note: FD: ‘Fuding Dabaicha’, CC: ‘CHuancha Erhao’, and CPI: combined parentage index.

## Data Availability

The relevant data obtained in this study have been submitted as Supplementary Materials.

## Supplementary Materials

The following supporting information can be downloaded at https://www.mdpi.com/article/10.3390/plants14223500/s1; Table S1. Information on 48 tea cultivars used for evaluating marker polymorphism. Table S2. The formula for calculating the paternity index (PI) based on Mendelian inheritance principles. Table S3. Genotype data of 48 tea cultivars at the 30 SSR marker loci. Table S4. Alleles (fragment size and corresponding repeat numbers) and allele frequencies of the 30 SSR markers. Table S5. Genotype data of the tested tea offspring at the 30 SSR marker loci.
